# The dendritic spine story: an intriguing process of discovery

**DOI:** 10.3389/fnana.2015.00014

**Published:** 2015-03-05

**Authors:** Javier DeFelipe

**Affiliations:** ^1^Instituto Cajal (CSIC)Madrid, Spain; ^2^Laboratorio Cajal de Circuitos Corticales (Centro de Tecnología Biomédica: UPM), and CIBERNEDMadrid, Spain

**Keywords:** pyramidal cells, granule cells, Purkinje cells, Cajal, Golgi, reticular theory, neuron doctrine, history of neuroscience

## Abstract

Dendritic spines are key components of a variety of microcircuits and they represent the majority of postsynaptic targets of glutamatergic axon terminals in the brain. The present article will focus on the discovery of dendritic spines, which was possible thanks to the application of the Golgi technique to the study of the nervous system, and will also explore the early interpretation of these elements. This discovery represents an interesting chapter in the history of neuroscience as it shows us that progress in the study of the structure of the nervous system is based not only on the emergence of new techniques but also on our ability to exploit the methods already available and correctly interpret their microscopic images.

## Introduction

In 1873, a revolution began in the world of neuroscience with the discovery by Camillo Golgi of a new technique to stain the nervous system which allowed neurons and glia to be visualized with all their processes and in great detail. Since these cells were labeled in black, Golgi referred to this technique as the “*reazione near*” (black reaction). Golgi was very enthusiastic about this discovery as reflected in a letter that he sent to his friend Niccolò Manfredi, where he outlined the new method (Mazzarello, 1999):

I spend long hours at the microscope. I am delighted that I have found a new reaction to demonstrate even to the blind the structure of the interstitial stroma of the cerebral cortex. I let the silver nitrate react with pieces of brain hardened in potassium dichromate. I have obtained magnificent results and hope to do even better in the future.

**Figure 1:**
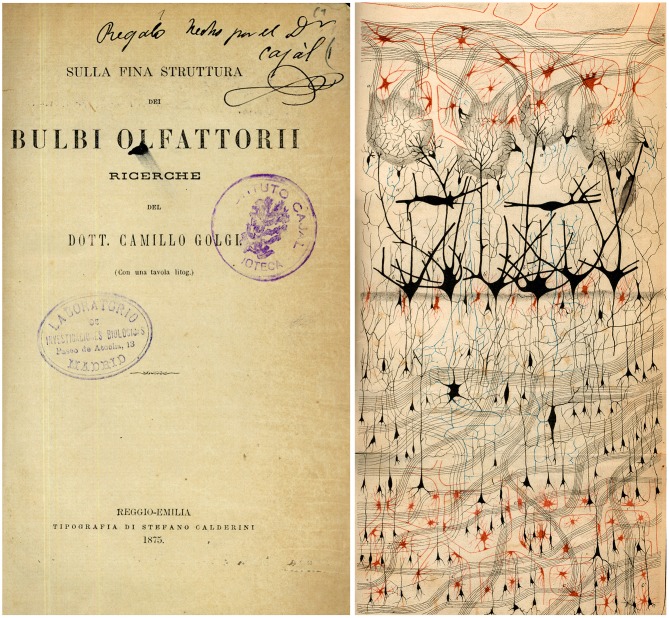
**The first illustration by Golgi of a Golgi impregnated preparation of the nervous system**. “*Semi-schematic drawing of a fragment of a vertical section of the olfactory bulb of a dog*” (Golgi, 1875). Taken from DeFelipe (2010).

**Figure 2:**
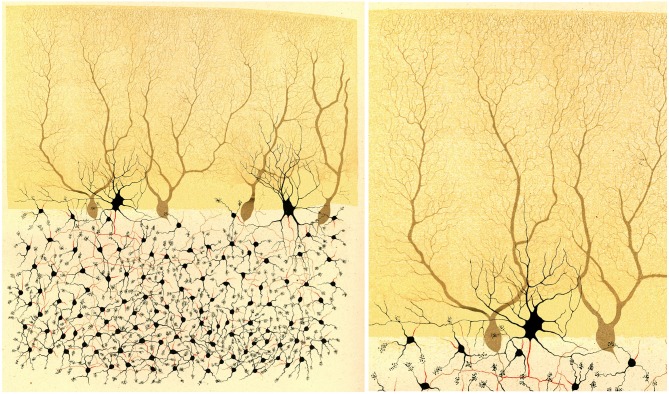
**Illustration by Golgi of a Golgi impregnated preparation of the cerebellum**. “*Fragment of a vertical section of a cerebellar convolution of the rabbit*”. *Left*, panoramic view. *Right*, a high magnification of the drawing to illustrate that the dendrites of the Purkinje cells are smooth, without dendritic spines. Taken from Golgi (1882–1883).

The method, named the Golgi method after its discoverer, was published in the *Gazzeta Medica Italiani* on the 2nd of August, 1873 (Golgi, 1873): *Sulla struttura della sostanza grigia del cervello* (On the structure of the gray substance of the cerebrum). Thanks to a very simple staining protocol, requiring a “prolonged immersion of the tissue, previously hardened with potassium or ammonium dichromate, in a 0.50 or 1.0% solution of silver nitrate”, it was possible for the first time to observe neurons and glia in a histological preparation (Figure 1) with all their parts (cell body, dendrites and axon, in the case of neurons; cell body and processes in the case of glia DeFelipe, 2002). Golgi used this method to examine many regions of the nervous system, providing new insights into the neuroanatomy of these structures and he illustrated the findings with beautiful drawings, as shown in Figures 1, 2. Figure 1 shows the organization of the olfactory bulb (nerve cells and pathways), whereas Figure 2 shows different types of neurons in the cerebellar cortex, in great morphological detail, albeit with some important exceptions, like the dendritic arborizations of Purkinje cells which appear free of dendritic spines (see below for further discussion on this).

The introduction of this method was a very important advance since, before this development, the visualization of neurons with the available histological techniques had been incomplete; it was only feasible to observe the cell body and the proximal portions of the dendrites and axon. Thus, it was not possible to follow the trajectory of the thin axons or to visualize the terminal axonal arbors as occurred with the Golgi method (Figures 1, 2). Indeed, at that time, the prevailing hypothesis about the organization of the nervous system was in fact the reticular theory, which proposed that the nerve cells of the nervous system formed a continuum, rather than existing as individual elements (later called the neuron doctrine). It was Joseph von Gerlach who really developed the reticular theory and thus, he is considered the father of this theory (von Gerlach, 1872). Interestingly, when Golgi examined the silver impregnated preparations, he concluded that the reticular theory supported by Gerlach was wrong, since Golgi thought that dendrites ended freely and that only the axons and their collaterals anastomose. Therefore, he suggested that the nervous system consisted of a “*rete nervosa diffusa*” (diffuse nervous network), an idea that he supported even as late as 1906 during his Nobel Price lecture. In contrast, Santiago Ramón y Cajal, using the same methods and microscopes as Golgi, strongly supported the neuron doctrine as he found that all processes of nerve cells end “freely” (i.e., nerve cells are individual elements) and that connections between them are by contact. Since Cajal contributed more than any other researcher of his time to support this theory, he is considered the father of the neuron doctrine (DeFelipe, 2002). As we will see below, dendritic spines of Purkinje cells and pyramidal cells were considered as good examples of transmission by contact.

The present article will focus on the discovery of dendritic spines, which was possible thanks to the application of the Golgi technique to the study of the nervous system, and will also explore the early interpretation of these elements. This discovery represents an interesting page in the history of neuroscience as it shows us that progress in the study of the structure of the nervous system is based not only on the introduction of new techniques but also on the ability to exploit the methods already available and the correct interpretation of their microscopic images. This review is largely based on several of my previously-published articles and books about the structure of the nervous system in the times of Cajal, and it has been divided into three sections: (1) First critical discoveries using the Golgi method: the neuron theory and visualization of dendritic spines; (2) Dendritic spines: true anatomical dispositions vs. artifacts; and (3) Other interpretations of the dendritic spines and final considerations.

## First critical discoveries using the Golgi method: the neuron theory and visualization of dendritic spines

Interestingly, for a long time after the discovery of the Golgi method in 1873, the method went virtually unnoticed. This was really unfortunate since this new powerful tool was practically not exploited by the scientific community and many discoveries had to wait for years to see the light of day. This situation was highlighted well by Cajal himself in *Recuerdos de mi vida* (Cajal, 1917):

I have already expressed above the surprise I felt when I saw with my own eyes the wonderful revelatory power of the chrome-silver reaction [Golgi method] and the indifference of the scientific community regarding this discovery. How could this disinterest be explained? Today, as I better understand the psychology of scholars, I find it very natural. In France, as in Germany, and more in the latter than in the former, a severe school discipline reigns. Out of respect for their master, it is common that disciples do not use research methods that have not been passed on by him. As for the great investigators, they would consider themselves dishonored if they worked with the methods of others.

Cajal was aware of the existence of the Golgi method, though he had not tested it since he did not think it was useful:

But, as I mentioned, the admirable method of Golgi was then (1887–1888) unknown to the immense majority of neurologists or was underestimated by the few who had precise information about it. Ranvier’s book, my technical bible of those days, devoted only a few descriptive lines of to it, written in an indifferent style. It was evident that the French savant had not tried it. Naturally, the readers of Ranvier, like myself, thought this method to be unworthy to be used.

Cajal’s interest in using this silver chromate method was thanks to Luis Simarro (1851–1921), a psychiatrist and neurologist who was also an enthusiast of histology. Interestingly, Simarro learned this method from Louis Antoine Ranvier (1835–1922), and introduced some modifications (Fernandez and Breathnach, 2001). It was in 1887 during a visit to Madrid that Cajal—who was living in Valencia at the time—was invited into Simarro’s own house, where he first saw a Golgi impregnated preparation (Cajal, 1917):

I owe to Luis Simarro the unforgettable favor of having been shown the first good preparations made by the method of silver chromate that I ever saw, and of his having called my attention to the exceptional importance of the book of the Italian savant devoted to the examination of the fine structure of the gray matter.

**Figure 3:**
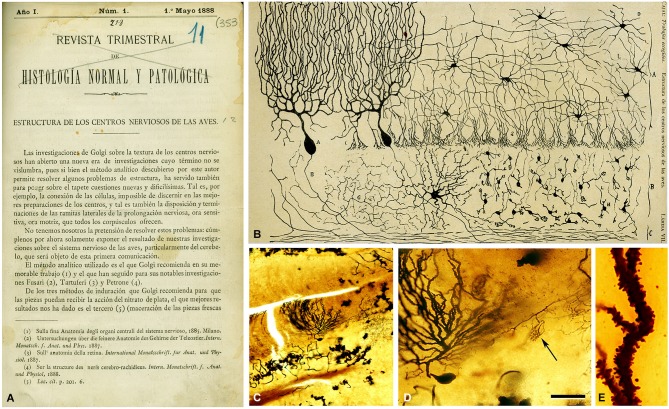
**First illustration by Cajal (1888) of a Golgi impregnated preparation of the nervous system. (A)** First page of the article and **(B)** illustration whose legend states: “*Vertical section of a cerebellar convolution of a hen. Impregnation by the Golgi method. A represents the molecular zone, B designates the granular layer and C the white matter*”. **(C)** photomicrograph from one of Cajal’s preparations of the cerebellum of an adult bird stained with the Golgi method. **(D)** higher magnification of **(C)** to illustrate a Purkinje cell and a basket formation (arrow). **(E)** dendrite of the Purkinje cell which is covered with dendritic spines. The histological images were obtained by Pablo García-López, Virginia García-Marín, and Miguel Freire (Legado Cajal, Instituto Cajal). Scale bar: 200 μm in **(C)**; 60 μm in **(D)**; 8,4 μm in **(E)**. Taken from DeFelipe (2014).

Cajal was captivated by this marvelous staining method and he immediately started to use it to analyze practically the entire nervous system in several species. One year after his meeting with Simarro, Cajal published his first important article based on results obtained with this method in the avian cerebellum (Figure 3). In this study entitled *Estructura de los centros nerviosos de las aves* (Cajal, 1888), Cajal made two great contributions:

-First, he confirmed Golgi’s conclusion that dendrites end freely but, in contrast to Golgi, Cajal added the decisive conclusion that this also applies to axons and their branches:

We have carried out detailed studies to investigate the course and connections of the nerve fibers in the cerebral and cerebellar convolutions of the human, monkey, dog, etc. We have not been able to see an anastomosis between the ramifications of two different nervous prolongations, nor between the filaments emanating from the same expansion of Deiters [axons]. While the fibers are interlaced in a very complicated manner, engendering an intricate and dense plexus, they never form a net […] it could be said that each [nerve cell] is an absolutely autonomous physiological canton [unit]

In the years that followed, he provided many examples from throughout the nervous system to support his observation that dendrites and axons end freely. The reticular and the neuron theories are obviously radically divergent regarding the interpretation of how nerve currents flow through a continuous rather than a discontinuous network of neuronal processes. Thus, the new ideas about the connections between neurons led to novel theories on the relationship between neuronal circuits and brain function. Cajal wrote in volume I of *Histologie du système nerveux de l’homme et des vertébrés* (Cajal, 1909–1911):

The cell bodies, [dendrites and axons] terminate freely but nevertheless, the flow of currents is not impeded in such an infinitely interrupted, fragmented nervous system. How can such currents flow? There can be only one answer, by contact, in much the same way that electric current crosses a splice between two wires.

These early studies with the Golgi method regarding the connections of neurons were so decisive that they represented the main core of the review published by Wilhelm von Waldeyer-Hartz in the journal *Deutsche Medizinische Wochenschrift* in 1891. In this article, the term “neuron” was introduced to denominate the nerve cells and the so-called neuron doctrine became popular. By the end of the XIXth century, this theory was the most accepted theory to explain the organization of the nervous system, in which the neuron was considered as the anatomical, physiological, genetic and metabolic unit of the nervous system. The many, fundamental contributions of Cajal to the neuron doctrine were summarized by himself in several articles and books, and especially in *¿Neuronismo o Reticularismo?* published in 1933 (Cajal, 1933). Cajal distinguished two main types of contacts between nerve cells: connections with the cell body and connections with the dendrites. In 1897, these contacts, also called “articulations” by Cajal, were baptized by Charles Sherrington (1857–1952) with the name of “synapses” (Foster and Sherrington, 1897). In his classic book *The Integrative Action of the Nervous System*, Sherrington masterfully described the hypothetical one-way contact between axon terminals and somata or dendrites, and the possible exceptions of the neuron theory (Sherrington, 1947):

As to the existence or non-existence of a surface of separation or membrane between neurone and neurone, that is a structural question on which histology might be competent to give valuable information. In certain cases, especially in Invertebrata, observation (Apathy, Bethe, etc.) indicates that many nerve-cells are actually continuous one with another. It is noteworthy that in several of these cases the irreversibility of direction of conduction which is characteristic of spinal reflex-arcs is not demonstrable […]. But in the neurone-chains on the gray-centred system of vertebrates, histology on the whole furnishes evidence that a surface of separation does exist between neurone and neurone. […] It seems therefore likely that the nexus between neurone and neurone in the reflex-arc, at least in the spinal arc of the vertebrate, involves a surface of separation between neurone and neurone; and this as a transverse membrane across the conductor must be an important element in intercellular conduction. […] In view, therefore, of the probable importance physiologically of this mode of nexus between neurone and neurone it is convenient to have a term for it. The term introduced has been *synapse* (Foster and Sherrington, 1897).

-Cajal’s second contribution was his description of the existence of dendritic spines (which he also named):

…the surface [of the dendrites of Purkinje cells] appears to be covered with thorns or short spines…(At the beginning, we thought that these eminences were the result of a tumultuous precipitation of the silver but the constancy of its existence and its presence, even in preparations in which the reaction appears to be very delicate in the remaining elements, incline us to believe this to be a normal condition).

However, in this article he did not discuss the possible function of dendritic spines. Two years later, Cajal described the existence of these structures in the pyramidal cells of the cerebral cortex and interpreted them as possible targets of axons (Cajal, 1890):

Layer of the small pyramids —We will only add two details to the description given by Golgi: first, the peripheral arborizations of the ascending shaft bristles with short spines ending in a small swelling. The gulfs that are between such collateral spines receive the impression of innumerable small fibers of the superficial layer. Exactly the same disposition is possessed by the terminal peripheral arborizations of the large pyramids.

It was not until 1892 that he more specifically referred to the dendritic spines as key elements for transmission by contact in both the cerebellar and cerebral cortices (Cajal, 1892). In this article, when referring to the connections between the axonal plexus of layer I with the ascending dendritic tufts of pyramidal cells ending in this layer, he wrote:

It is impossible not to consider this singular arrangement, which, by the way, is found with the same characteristics in all vertebrates, as an important example of neural transmission by contact, comparable to that occurring in the cerebellum between the tiny parallel fibers and the [dendritic] arborizations of the Purkinje cells. This contact would be transverse or oblique, on account of which the terminal [dendritic] branches of the pyramidal cells possess short collateral spines, in the gaps between which the thinnest small axonal fibers bereft of myelin seem to be tightly caught.

**Figure 4:**
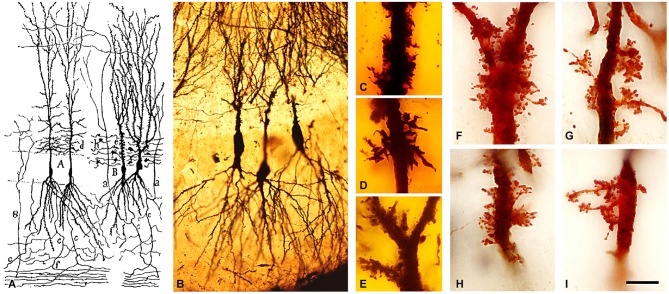
**Complex dendritic spines (thorny excrescences) of CA3 pyramidal neurons. (A)** Cajal’s drawing showing pyramidal cells with thorny excrescences in the CA3 (Cajal, 1893). **(B–I)** photomicrographs of Cajal’s original histological preparations housed at the Cajal Institute. **(B)** CA3 pyramidal cells of the rabbit stained by the Golgi method. **(C–I)** Examples of thorny excrescences on CA3 pyramidal neurons. **(C–E)** Dendrites from a newborn child’s CA3 pyramidal neurons stained by the Golgi method; **(F–I)** Dendrites of rabbit CA3 pyramidal neurons stained by Kenyon’s variant of the Golgi method. Scale bar: **(B)** 55 μm;** (C–I)**, 11 μm. Taken from Blazquez-Llorca et al. (2011).

Furthermore, in 1893, he also discovered and named the typical thorny excrescences of hippocampal pyramidal cells of CA3 (Cajal, 1893). He proposed that these large and often branched structures served as points of contact with the mossy fibers from the dentate gyrus (Figure 4), an observation that is well established at present (Andersen et al., 2007). In addition, Cajal observed in the immature nervous system the presence of short dendritic protrusions that were different to dendritic spines and that he described as “irregular projections very seldom ending as bulbs” and that were most likely transitory (Cajal, 1933). Later, these transitory protrusions were called “filopodia”. After these studies, numerous researchers confirmed the existence of spines (e.g., Retzius, 1891; Schaffer, 1892; Edinger, 1893; Demoor, 1896; Stefanowska, 1897, 1901; Hatai, 1903). Among the later researchers, it is interesting to highlight the studies of Micheline Stefanowska—one of the very few female neuroscientists of that time. Her work was based on the 1890s hypothesis of amoeboid movement of neurons to explain possible changes in brain circuits as plastic responses of the normal brain or after experimental manipulation. For example, it was proposed that inactivity during sleep could be explained by a retraction of dendritic processes (theory of the histophysiology of sleep). This retraction would considerably reduce the number of contacts and thus “the association of the individual cellular activities” (DeFelipe, 2006). Stefanowska focused on the “piriform appendages”, the name used by her to refer to the dendritic spines. She proposed that these piriform appendages could temporarily change both their shape and number in the brain during normal activity and that similar changes may occur in the brain of experimental animals under certain experimental conditions such as electrical stimulation of the cerebral cortex or after the administration of sedatives (morphine, chloral hydrate and chloroform), etc. In general, the conclusions regarding the activity-dependent changes in dendritic spines have received particular attention again in recent times as many of these ideas have been confirmed with modern techniques (e.g., DeFelipe, 2006; Yuste, 2010).

## Dendritic spines: true anatomical dispositions vs. artifacts

**Figure 5:**
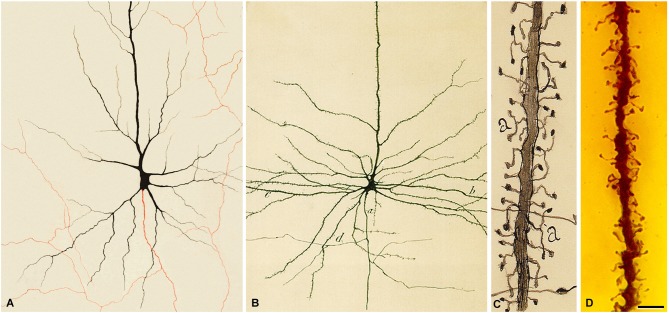
**Pyramidal cells of the human cerebral cortex. (A)** Drawing made by Golgi to illustrate a pyramidal cell of the human motor cortex stained with the Golgi method. The axon appears in red. Taken from Golgi (1882–1883). **(B)** Drawing made by Cajal to illustrate a pyramidal cell of the human motor cortex. *a*, initial part of the axon; *b*, dendrites; *d*, axonal collaterals. Taken from Cajal (1899). **(C)** Drawing by Cajal to illustrate the dendritic spines of pyramidal cells (cerebral cortex of a 2-month-old child). Taken from Cajal (1933). Note that Golgi does not draw dendritic spines. However, in the drawing of Cajal shown in **(B)** it can be seen that the surface of the dendrites are covered with dendrites spines. **(D)** photomicrograph of a preparation of Cajal of the human motor cortex (15-day-old child) stained using the Golgi method. The image illustrates an apical dendrite of a layer V pyramidal cell covered with spines. Scale bar (in **D**): **8** μm. The histological image was obtained by Pablo García-López, Virginia García-Marín and Miguel Freire (Legado Cajal, Instituto Cajal). Taken from DeFelipe (2014).

However, for some time, other prestigious authors such as Kölliker (1896), and even Golgi himself, considered that dendritic spines were artifacts, like needle-shaped crystallizations of silver chromate on the surface of neurons, and therefore these structures were not included in their early drawings and consequently dendrites appeared smooth (Figure 5A). This skepticism was not only due to the different interpretation of the microscopic images, as shown in Figure 5, but also because, at that time, dendritic spines were visualized only with the Golgi method or with a variant of this method, the Golgi-Cox method (Cox, 1891: tissue samples are immersed in a mixture of potassium dichromate and mercuric chloride). Furthermore, in 1895 Meyer published an article reporting that by using a variant of the methylene blue technique he could not verify the existence of these structures (Meyer, 1895, 1896, 1897). The methylene blue technique was introduced by Ehrlich (1881) as a bacteriological stain (Ehrlich, 1881) but it was in 1886 that Ehrlich found that peripheral nerve fibers could be stained by injecting the stain into the blood vessels of living animals (Ehrlich, 1886). This approach became popular as it rendered images comparable to those obtained with Golgi impregnation. The methylene blue procedure was modified by several authors, in particular Bethe (1895) who introduced fixation with ammonium molybdate to improve the preservation of the staining during the various steps of histological processing of the sections, and Dogiel (1896) who introduced some practical modifications allowing the application of the technique to thin, freshly-cut slices of central nervous tissue. Since dendritic spines were consistently stained with both the method of Golgi and with the variant of Cox, in specific regions of the neurons, Cajal interpreted this as clear evidence of their existence, and concluded that therefore dendritic spines must be key elements in the structure and function of neurons. Cajal (1896) noted:

…Dendritic spines are constantly present at the same regions of the [dendritic] arborizations, no matter what animal is studied, and are always lacking at certain sites, such as the [axon initial segment], cell body and origin of the thick [dendritic] processes.

**Figure 6:**
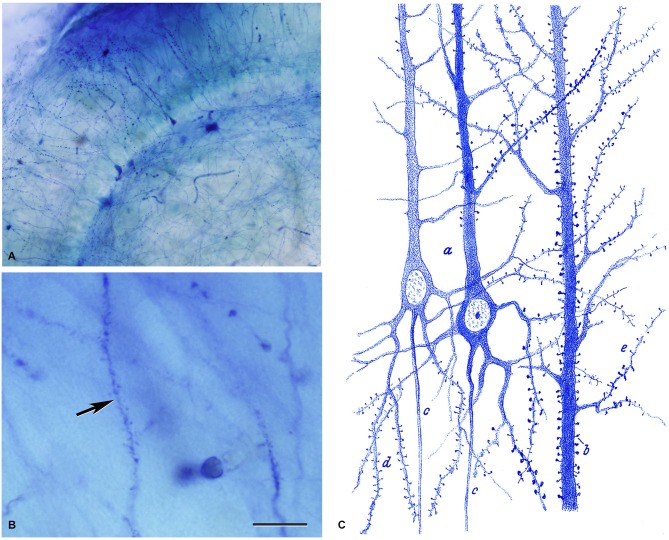
**Dendritic spines stained with the methylene blue method. (A,B)** Low and high magnification photomicrographs, respectively, of a preparation by Cajal of the hippocampus stained with the methylene blue method (preparation housed at the Cajal Institute). These photomicrographs are from unpublished material from DeFelipe and Jones (1988). Scale bar (in **B**): **(A)** 120 μm;** (B**) 10 μm.** (C)** Drawing used by Cajal to show the existence of dendritic spines on pyramidal cells with the methylene blue method. Taken from Cajal (1899–1904).

However, due to the contradictory results of Meyer and the fact that Kölliker, who was much admired by Cajal, also denied the existence of dendritic spines, Cajal was prompted to re-examine this topic using the methylene blue method. Cajal used the same methodology as Meyer did and found that dendrites were stained so pale that their spines were not visible and therefore, the method applied by Meyer was inappropriate to show their existence. Cajal then modified the methylene blue procedure based on the procedures of Dogiel and Bethe, and found that dendritic spines were also stained with this method (Cajal, 1896; Figure 6). In addition, other authors confirmed the visualization of dendritic spines with the methylene blue method (e.g., Turner and Aber, 1900; Soukhanoff et al., 1904) and, therefore, it was concluded that they were not artifacts, but actual anatomical arrangements.

**Figure 7:**
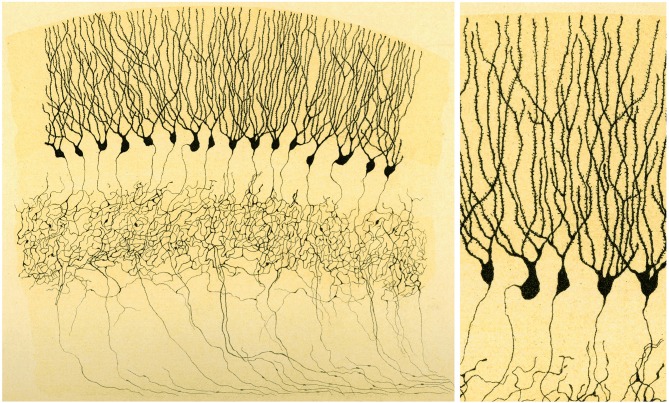
**Illustration by Golgi of a Golgi impregnated preparation of the dentate gyrus**. “*Fascia dentata del grande piede di Hippocampo”*. **Left**, panoramic view. Golgi used this drawing to illustrate that the axons of granule cells formed a very complex nervous network (“*rete nervosa*”). **Right**, High magnification of the drawing to illustrate that Golgi recognized the presence of dendritic spines. Taken from Golgi (1901).

Interestingly, Golgi went on to publish an article (in 1901) where he drew dendritic spines (Figure 7). To my knowledge, it is the first time that he recognized the existence of these structures (Golgi, 1901). However, in the text he does not mention the dendritic spines. Therefore, it is clear that Golgi recognized the existence of dendritic spines, probably after the new evidence obtained with the methylene blue method used by Cajal and others scientists, but it seems that Golgi did not attribute any functional relevance to these structures.

## Other interpretations of the dendritic spines and final considerations

**Figure 8:**
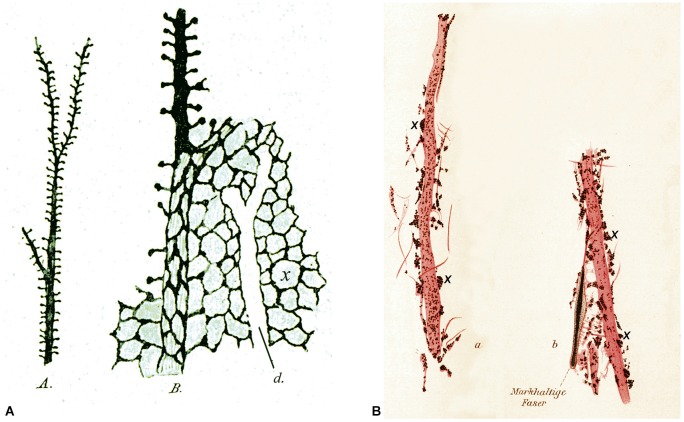
**Different interpretation of dendritic spines. (A)** Drawings made by Bethe (1903) to illustrate dendritic spines stained with the ammonium molybdate method. *A*, an apical dendrite of a pyramidal cell covered with dendritic spines. *B*, schematic representation to illustrate his hypothesis that dendritic spines were the starting points of an interstitial network of the gray matter. *d*, unstained apical dendrite. **(B)** Drawings made by Held (1897b) to show dendrites innervated by end-feet or axon terminals (some of them are marked with an x). See text for further details.

Another interesting aspect of the early history of dendritic spines is that some authors confirmed their existence, but they proposed or interpreted their functions differently from Cajal. Among the most representative examples of these postulated functions are those put forward by the distinguished neuroscientists Bethe and Held (Figure 8). Bethe (1903) drew dendritic spines as Cajal did, but he proposed that they were the initial points of an interstitial network of the gray matter that Nissl named *nervöse Grau* (Figure 8A). Held recognized the existence of dendritic spines but he proposed that they represented the genuine end-feet (axons ended in distinct terminals) or *Endfüsse* that were incompletely stained or fragmented (Figure 8B). He thought that these axon terminals were fused with the dendrites, as occurred with the end-feet on the cell bodies (Held, 1897a,b, 1904, 1905, 1929). In addition, Held hypothesized the existence of an interstitial network that would allow communication between pyramidal cells thanks to the anastomoses between these “dendritic spines” and the nerve fibers (Held, 1929).

**Figure 9:**
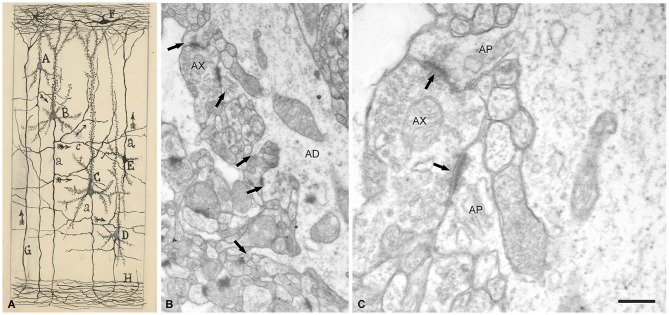
**Drawing and images showing dendritic spines as postsynaptic structures. (A)** Schematic drawing by Cajal to show synaptic connections and the possible flow of information through neural circuits in the cerebral cortex. The legend states: *A, small pyramid; B, and C, medium and giant pyramids, respectively; a, axon; [c], nervous collaterals that appear to cross and touch the dendrites and the trunks [apical dendrites] of the pyramids; H, white matter*; [*E*, Martinotti cell with ascending axon]; *F, special cells of the first layer of cerebral cortex; G, fiber coming from the white matter. The arrows mark the supposed direction of the nervous current”*. Taken from *¿Neuronismo o reticularismo?* (Cajal, 1933). **(B)** Electron micrograph of a typical apical dendrite showing dendritic spines with different shapes (arrows). **(C)** high magnification of **A** to illustrate an axon terminal (*AX*) which establishes synaptic contacts (arrows) simultaneously with two dendritic spines. AP, spine apparatus. Scale bar: **(B)** 0,60 μm; **(C)** 0,15 μm. Taken from unpublished material (Alonso-Nanclares et al., 2008).

Cajal paid special attention to this publication and aimed to discredit it, driven by being at odds with the interpretation of Held, as well as by the fact that Held had wrongly attributed the discovery of dendritic spines to Golgi. Thus, in *¿Neuronismo o reticularismo?* (Cajal, 1933), Cajal presented a strong argument against the interpretation by Held basing his criticism on the fact that with the neurofibrillar methods the finest axon terminals are visualized, whereas the dendritic spines of pyramidal cells or Purkinje cells are never stained. To make this point clearer, in this article Cajal included an elegant schematic drawing (Figure 9A) in which the connections by contact of the collateral axonal branches of the pyramidal cells with dendritic spines were highlighted after analyzing the “extremely complex, diffuse nerve plexuses” of the cerebral cortex:

I referred particularly to this intricate plexus when I lamented the insurmountable difficulties facing the analysis of the cortical synapses […]. Note how these collaterals cross and enter into transversal or oblique contact with a great number of the dendritic shafts. It is probable that collaterals rest on the spines which cover the protoplasmic surfaces [dendrites] like down .... I have never seen anastomoses between the spines and the nerve fibers, despite having devoted particular attention to them since 1888.

**Figure 10:**
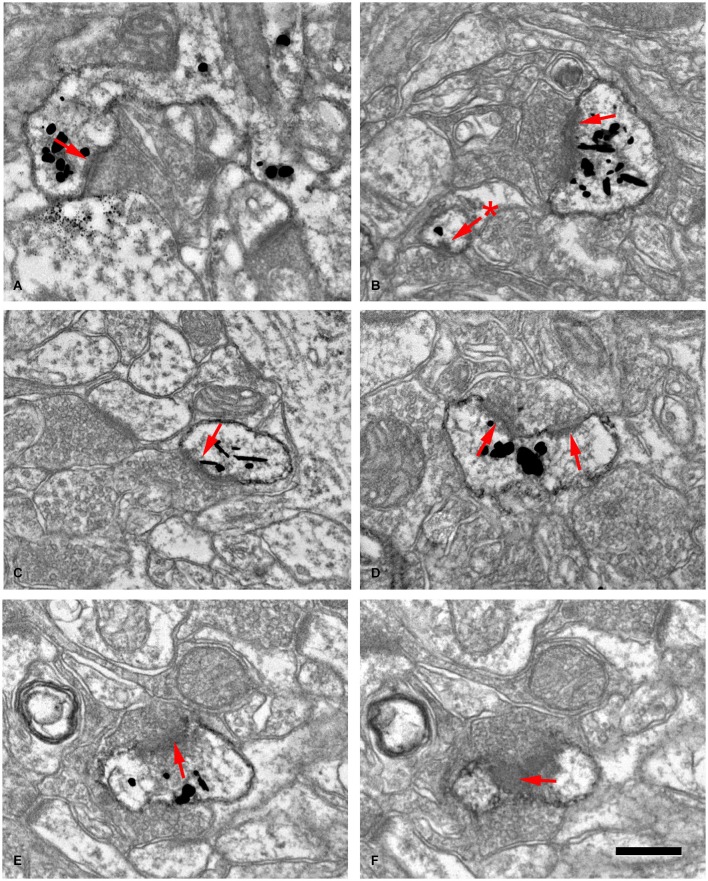
**Examples of dendritic spines forming synapses (A–F) in the adult mouse neocortex**. The gold-particles allow the postsynaptic densities (PSDs; red arrows) to be clearly distinguished when present. Note the small size of the postsynaptic density (60 nm) in the head of a small spine in **(B)** (asterisk). **(E,F)** are consecutive sections of a spine head to illustrate a PSD cut tangentially. Scale bar: 560 nm in **(A)**; 350 nm in **(B,C)**; 300 nm in **(D)**; 280 nm in **(E,F)**. Taken from Arellano et al. (2007).

**Figure 11:**
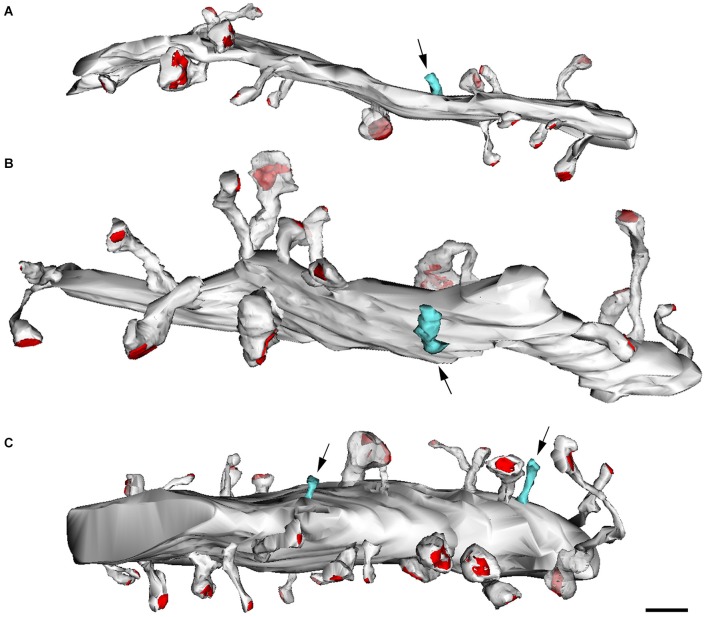
**Reconstructions from serial electron micrographs.** Serial sections of dendritic segments in the adult mouse neocortex to illustrate the distribution of some non-synaptic spines (blue) indicated by arrows. The remaining spines establish synaptic contacts (red, PSD). **(A,B)** Basal dendrites; **(C)** apical dendrite. Scale bar: 2000 nm. Taken from Arellano et al. (2007).

Thus, after discovering dendritic spines, Cajal proposed that these elements established connections with axon terminals, and in his last articles he sometimes used the term “synapses” to refer to these connections (e.g., Cajal, 1933). Nevertheless, the advent and development of electron microscopy in the 1950s to study the nervous system (Robertson, 1953; Palade and Palay, 1954; De Robertis and Bennett, 1955; Palay, 1956; De Robertis, 1959) was necessary for the confirmation that they were really postsynaptic structures (Figure 9). The first electron microscope study of dendritic spines showing that they establish synaptic connections was published by Gray (1959), and these observations were confirmed and extended by several authors (e.g., Jones and Powell, 1969; Peters and Kaiserman-Abramof, 1969, 1970). Nevertheless, direct demonstration that the dendritic spines of Golgi-impregnated neurons established synapses came with the introduction of the combination of the Golgi method and electron microscopy, particularly the gold-toning technique of Fairén and colleagues (Fairén et al., 1977). This method involves de-impregnation of previously Golgi-impregnated neurons, followed by the study of their fine structure by electron microscope. The original impregnation deposit of silver chromate (which produces an intense, homogeneous intracellular labeling that masks postsynaptic densities) is replaced by a deposit of gold particles. At the electron microscope level, the deposit is visible as fine particles that mark the profiles of the de-impregnated neurons while allowing the visualization of the cytological details of the labeled neuron. Thus, this technique enables the accurate study of the ultrastructural characteristics and synaptic connections of Golgi-impregnated neurons, including dendritic spines, as the postsynaptic densities can be clearly distinguished (Figure 10). Further development of combinations of a variety of techniques (degeneration methods, immunocytochemistry, etc.,) for correlative light and electron microscopy soon followed. The application of these methods allowed the detailed examination of the afferent and efferent connections and chemical characteristics of Golgi-impregnated neurons (e.g., Frotscher and Léránth, 1986). Indeed, at present it is well established that almost all dendritic spines establish at least one excitatory glutamatergic synapse, as only a small portion of dendritic spines have been found to be non-synaptic (Figure 11; Arellano et al., 2007). Finally, the collective work of numerous authors have shown that dendritic spines are the major targets of excitatory connections in the cerebral cortex and that they seem to be key elements in learning, memory, and cognition (Yuste, 2010).

## Conflict of interest statement

The author declares that the research was conducted in the absence of any commercial or financial relationships that could be construed as a potential conflict of interest.
